# INADEQUATE PHYSICAL ACTIVITY ON HEALTHCARE EXPENDITURES AMONG MIDDLE AGE AND OLDER WORKING ADULTS IN THAILAND

**DOI:** 10.1093/geroni/igac059.3060

**Published:** 2022-12-20

**Authors:** Katika Akksilp, Wanrudee Isaranuwatchai, Yot Teerawattananon, Cynthia Chen

**Affiliations:** National University of Singapore, Singapore, Singapore; Health Intervention and Technology Assessment Program, Muang Nonthaburi, Nonthaburi, Thailand; Health Intervention and Technology Assessment Program, Muang Nonthaburi, Nonthaburi, Thailand; National University of Singapore, Singapore, Singapore

## Abstract

Physical inactivity is a significant risk factor for developing non-communicable diseases. This study estimates the additional health costs due to physical inactivity in Thai middle age and older working adults. We included participants aged 40 years and above from the Physical Activity at Work study, who had valid physical activity data from ActiGraph. Health costs were collected using the Health and Welfare Survey and the Work Productivity and Activity Impairment Questionnaire in Thailand. Direct and indirect costs (absenteeism and presenteeism) of the most recent illness in the previous month were collected. Active participants were defined as having at least 150 minutes of moderate-intensity equivalent activity per week. Two-part models were used to compare health costs between active and non-active participants. Data from 105 participants aged 40 to 63 were included in the analysis, where 42% of participants were active. Inadequate physical activity was significantly associated with increased direct health costs of 160.5THB (95%CI: 31.3 to 289.7THB) (~7.8 times higher in inactive). Further adjustment for sex, age, education, obesity, and cardiovascular condition yielded similar findings. After incorporating these indirect costs, the societal cost of having inadequate physical activity was 791.22THB (95%CI: 115.6 to 1466.8THB) (~1.3 times higher in inactive), even after adjusting for the covariates. Obesity and the female were also associated with higher societal costs (551.9THB and 342.9THB, respectively), although these results were not significant. Increasing physical activity in middle age and older working adults to meet the current guidelines may reduce Thailand’s health costs.

